# Sociodemographic characteristics and transmission risk factors in patients hospitalized for COVID-19 before and during the lockdown in France

**DOI:** 10.1186/s12879-021-06419-7

**Published:** 2021-08-13

**Authors:** Mayda Rahi, Diane Le Pluart, Alexandra Beaudet, Sophie Ismaël, Marion Parisey, Nora Poey, Hassan Tarhini, François-Xavier Lescure, Yazdan Yazdanpanah, Laurène Deconinck

**Affiliations:** Infectious and Tropical Diseases Department|, Bichat - Claude-Bernard University Hospital, AP-HP, 46 rue Henri Huchard, 75018 Paris, France

**Keywords:** COVID-19, Lockdown, Sociodemographic characteristics

## Abstract

**Background:**

The efficacy of lockdown in containing the COVID-19 pandemic has been reported in different studies. However, the impact on sociodemographic characteristics of individuals infected with SARS-CoV-2 has not been evaluated. The aim of this study was to describe the changes in sociodemographic characteristics of patients hospitalized for COVID-19 and to compare the transmission risk factors of COVID-19 before and during lockdown in France.

**Methods:**

An observational retrospective study was conducted in a University Hospital in Paris, France. Data from patients hospitalized for COVID-19 in the Infectious Diseases Department between February 26 and May 11, 2020 were collected. The study population was divided into 2 groups: group A of patients infected before lockdown, and group B of patients infected during lockdown, considering a maximum incubation period of 14 days. Sociodemographic characteristics and transmission risk factors were compared between the 2 groups using Student’s t-test for continuous variables and Chi-2 test or Fisher exact test for categorical variables.

**Results:**

Three hundred eighty-three patients were included in the study, 305 (79.6%) in group A and 78 (20.4%) in group B. Patients in group A were significantly younger (60.0 versus (vs) 66.5 years (*p* = 0.03)). The professionally active population was larger in group A (44.3% vs 24.4%). There were significantly more non-French-speaking people in group B (16.7% vs 6.6%, *p* <  0.01). Most patients from group A had individual accommodation (92.8% vs 74.4%, p <  0.01). Contact with a relative was the main transmission risk factor in both groups (24.6% vs 33.3%, *p* = 0.16). Recent travel and large gathering were found only in group A. The proportion of people living in disadvantaged conditions, such as homeless people or people living in social housing, was significantly higher in group B (11.5% vs 4.3%, *p* = 0.03) as was the proportion of institutionalized individuals (14.1% vs 3.0%, *p* <  0.01).

**Conclusions:**

In this study conducted in patients hospitalized for COVID-19 in Paris, France, the likelihood of being infected despite the lockdown was higher for people who do not speak French, live in social housing, are homeless or institutionalized. Targeted measures have to be implemented to protect these populations.

## Background

The COVID-19 pandemic is an unprecedented public health challenge. In the past century, dramatic changes in demographics, urbanization and globalization have facilitated the spread of infectious diseases across countries and continents. In a matter of weeks, the COVID-19 pandemic devastated communities worldwide, requiring a multinational governmental, healthcare system and public health response [1].

The outbreak of COVID-19 was declared as a global pandemic on March 11, 2020 and by May 30, 2020, about 6 million positive cases were registered worldwide [2]. As in many countries facing the pandemic, restrictive measures were progressively implemented in France from February 2020, such as a ban on public gatherings in enclosed spaces, closure of nurseries, schools and universities, physical distancing, and the use of face masks. Due to the continuously increasing incidence despite all the above measures and in order to help the healthcare system cope with the ever higher number of patients, the French Government declared, on March 17, a general population lockdown, as well as travel restrictions in the European Union and border closure of the Schengen area. The entire French population was in strict lockdown until May 11.

The efficacy of lockdown in containing the pandemic has been predicted by mathematical modeling [3]. Lockdown has also been reported to reduce the number of new cases in countries that implemented it [4] and to decrease the spread of COVID-19 in areas where there was general lockdown of the entire population, such as Wuhan in China and Asian countries [5]. The efficacy of lockdown is especially noticed around 10 days from its implementation and continues to grow until 3 weeks [4].

However, the impact of lockdown on the sociodemographic characteristics of individuals infected with SARS-CoV-2 has not been well evaluated. It is known that socioeconomic position is a determinant of infectious diseases [6]. The more disadvantaged people are probably more likely to present with main risk factors for developing COVID-19. Therefore, it is capital to consider socioeconomic characteristics to identify the high-risk groups for transmission of COVID-19, especially during the lockdown [6].

The aim of this study was to describe the changes in sociodemographic characteristics of patients hospitalized for COVID-19 in a University Hospital in Paris and to compare the transmission risk factors of COVID-19 before and during lockdown in France.

## Methods

### Study design and population

In this observational, retrospective, single-center study, we included all confirmed cases of COVID-19 between February 26 and May 11, 2020 admitted to the Infectious Diseases Department of Bichat-Claude Bernard University Hospital in Paris, France. All enrolled patients were diagnosed by positive reverse-transcription polymerase chain reaction for SARS-CoV-2 and/or typical chest computerized tomography characteristics. The study population was divided into 2 groups: group A of patients infected before lockdown, and group B of patients considered to be infected during lockdown. A maximum incubation period of 14 days was considered as suggested by previous studies [7, 8]. Therefore, group A consisted of patients with onset of infection between February 26 and March 31, 2020. Group B consisted of patients complaining of initial signs from April, 1 until May 11, 2020.

### Outcomes and variables

The sociodemographic characteristics of patients hospitalized for COVID-19 before and during lockdown and the change in transmission risk factors between the 2 groups were collected. Sociodemographic data included age, gender, nationality, spoken language, occupation, healthcare insurance, type of household, and number of household members.

The potential transmission risk groups were assessed for each patient and classified in 8 categories as follows: (1) travel in an endemic international zone or in a cluster zone in France (city of Mulhouse, Oise department, Morbihan department); (2) unusual recent large gathering; (3) profession considered at risk (healthcare worker, caregiver, or public transport agent); (4) recent contact with a relative at high risk of infection (such as healthcare worker), presenting signs of infection or with confirmed COVID-19; (5) disadvantaged conditions (collective housing and homeless people); (6) nosocomial exposure (hospitalization and hemodialysis session); (7) institutionalized (elderly people and disabled persons); (8) not belonging to these transmission risk groups. Patients could belong to multiple transmission risk groups.

### Data collection and analysis

Data collection was done using computerized medical records (Orbis® software). We retrospectively analysed the medical charts records of every patient during hospitalization.

A standardized and anonymized questionnaire was used in order to screen the charts and verify the diagnosis based on the inclusion criteria, the onset of infection and to collect the different sociodemographic characteristics and potential transmission risk factors in every patient; the questionnaire was filled and revised by two medical physicians for validation.

Statistical analyses were performed using Stata Version 12.0. Continuous variables were described by median and interquartile ranges, whereas categorical variables were represented by numbers and percentages. Student’s t-test was used to compare continuous variables. The Chi-2 test or Fisher exact test was used to compare categorical variables according to the distribution and headcounts of variables. A *p*-value < 0.05 was considered statistically significant.

## Results

Sociodemographic characteristics of patients and transmission risk groups before and during lockdown are reported in Table 1. Sociodemographic characteristics and transmission risk factors before and during lockdown.

**Table 1:** Sociodemographic characteristics and transmission risk factors before and during lockdown

	Total	Infected before lockdown	Infected during lockdown	p
Total population, N (%)	383 (100.0)	305 (79.6)	78 (20.4)	
Demographic characteristics				
Men, n (%)	237 (61.9)	187 (61.3)	50 (64.1)	0.65
Age (median ± IQR)	61 (39–83)	60 (39–81)	66.5 (43–90)	0.03
Nationality, n (%)				
French	234 (61.1)	188 (61.6)	46 (59.0)	
European	18 (4.7)	13 (4.3)	5 (6.4)	
Non-European	131 (34.2)	104 (34.1)	27 (34.6)	0.65
Social characteristics				
Occupation, n (%)				
Active*	154 (40.2)	135 (44.3)	19 (24.4)	
Unemployed	51 (13.3)	40 (13.1)	11 (14.1)	
Retired	136 (35.5)	100 (32.8)	36 (46.2)	
Other or undetermined	42 (11.0)	30 (9.8)	12 (15.4)	0.01
Housing, n (%)				
Individual accommodation	341 (89.0)	283 (92.8)	58 (74.4)	
Social	15 (3.9)	10 (3.3)	5 (6.4)	
Homeless	7 (1.8)	3 (1.0)	4 (5.1)	
Institutionalized	20 (5.2)	9 (3.0)	11 (14.1)	< 0.01
Number of persons in the household, n (%)				
1	67 (17.5)	56 (18.4)	11 (14.1)	
2–4	199 (52.0)	164 (53.8)	35 (44.9)	
≥ 5 or collective housing	90 (23.5)	73 (23.9)	17 (21.8)	0.92
Non-French-speaking, n (%)	33 (8.6)	20 (6.6)	13 (16.7)	< 0.01
No health insurance, n (%)	24 (6.3)	18 (5.9)	6 (7.7)	0.60
Transmission risk factors				
Relative, n (%)	101 (26.4)	75 (24.6)	26 (33.3)	0.16
Nosocomial exposure, n (%)	44 (11.5)	35 (11.5)	9 (11.5)	0.99
Profession at risk, n (%)	40 (10.4)	33 (11.1)	7 (9.0)	0.79
Travel, n (%)	30 (7.8)	30 (9.8)	0 (0.0)	< 0.01
Disadvantaged conditions, n (%)	22 (5.7)	13 (4.3)	9 (11.5)	0.03
Institutionalized patients, n (%)	20 (5.2)	9 (3.0)	11 (14.1)	< 0.01
Large gathering, n (%)	10 (2.6)	10 (3.3)	0 (0.0)	0.22
No evident cause of infection, n (%)	136 (35.5)	120 (39.3)	16 (20.5)	< 0.01

*Active: employee/self-employed/executive

### Sociodemographic characteristics

Three hundred and eighty-three patients were included in the study, 305 (79.6%) in group A and 78 (20.4%) in group B. Patients in group A were significantly younger, with a median age of 60.0 years old (IQR 39–81) versus (vs) 66.5 years old (IQR 43–90) in group B (*p* = 0.03). Occupation was significantly different between the 2 groups (*p* <  0.01). The professionally active population was indeed larger in group A (44.3% vs 24.4%), while the percentage of retirees was higher in group B (46.2% vs 32.8%). However, unemployed people were equally distributed in the 2 groups (13.1% vs 14.1%). Patients were mainly of French nationality, 61.6 and 59.0% in groups A and B, respectively. The proportion of French, European, and non-European citizens was equally distributed in the 2 groups (*p* = 0.65). However, there were significantly more non-French-speaking people in group B compared to group A (16.7% vs 6.6%, *p* < 0.01). Regarding housing, most patients (92.8%) from group A had individual accommodation vs 74.4% in group B, whereas there were more people living in social housing (6.4% vs 3.3%), homeless people (5.1% vs 1.0%), and institutionalized individuals (14.1% vs 3.0%) in group B (*p* < 0.01). Healthcare coverage was not significantly different between the two groups, with 5.9 and 7.7% of the population with no health insurance in groups A and B, respectively (*p* = 0.60).

### Transmission risk groups

Contact with a relative as defined above was the predominant factor reported regardless of the period of infection (24.6% vs 33.3%, *p* = 0.16). Recent travel was only found in group A (9.8% vs 0.0%, *p* < 0.01), as was attendance at a recent large gathering (3.3% vs 0.0%, *p* = 0.22). The proportion of people with a profession considered at risk was equally distributed in the 2 groups (11.1% vs 9.0%, *p* = 0.79). In group B, the proportion of people living in disadvantaged conditions was significantly higher (11.5% vs 4.3%, *p* = 0.03) as was the proportion of institutionalized patients (14.1% vs 3.0%, *p* < 0.01). The nosocomial infection rate was the same in the 2 groups (11.5 and 11.5%, *p* = 0.99). Not belonging to a transmission risk group was more frequent in group A (39.3% vs 20.5%, *p* < 0.01).

## Discussion

In this study, lockdown effectively reduced the number of new infections, with 80% of hospitalized patients being infected before its implementation. The same result has been observed in other countries both in Asia and Europe [5, 9] and reflects a high level of adherence to lockdown in these populations. A recent observation comparing countries from all over the world demonstrated a significant reduction in COVID-19 new cases in countries that implemented a general lockdown comparing to those that did not [4]. In our study, the protective effect of lockdown was particularly notable in younger, professionally active people living in individual accommodation, probably because of the major effect of lockdown on their day-to-day life.

However, our results indicate that some populations remained at risk of infection despite lockdown. First, ethnic minorities represented one-third of the patients hospitalized with COVID-19 in both periods and individuals not speaking French were at higher risk of infection during lockdown. In fact, pandemics rarely affect all people in a uniform way such as in the Black Death in the fourteenth century in which the highest number of deaths was observed among the poorest populations. Inequitable conditions and response to COVID-19 are also being seen in many countries [10]. Previous studies in the United-Kingdom have reported a higher risk of COVID-19 infection in black and Asian people than in white people [11, 12]. Ethnic minorities may have limited access to information from health authorities and less knowledge about the disease. This misinformation and miscommunication may lead ignorance of government health warning and thus to inappropriate behaviors [10, 13, 14]. Second, people living in social housing and homeless people were more likely to be infected during lockdown. A higher risk of infection in more deprived areas has also been reported in previous studies [11, 12]. A seroprevalence study conducted by Médecins Sans Frontières in food distribution sites, emergency shelters, and workers’ residences in Paris and its suburbs in June 2020 revealed that COVID-19 seropositivity reached 52%, with a strong association with overcrowding [15]. The limited access to COVID-19 screening tests and health services affects more profoundly the poorest populations especially during times of crisis [10]. In many studies, social features of health such as poverty, homelessness and ethnicity are proven to have considerable effect on COVID-19 transmission and outcomes. A higher risk of viral transmission is noted in homeless families because of their crowded living spaces and the absence of efficient social distancing in this disadvantaged conditions [10, 14, 16, 17].

In addition, individuals infected during lockdown were older and included more retirees and institutionalized persons. This population is more dependent and requires constant care, leading to a risk of infection by caregivers [18]. Implementation of social distancing is challenging in crowded long-term care facilities with shared common areas and low preparedness for infection control [19]. Therefore, long-term care facilities should monitor actively and rapidly isolate potentially infected patients as well as healthcare workers and limit access to visitors during the escalating COVID-19 outbreak among their vulnerable patients [20].

In our study, the number of individuals without any identified risk factor for transmission was high, and even higher before lockdown, when the virus was circulating and the general population was unaware of its route of transmission.

Our patients were mostly infected by a relative before and during lockdown. Mathematical modeling of the effect of lockdown in Italy showed that even with strict adherence to lockdown, transmission will still occur within households [3]. Physical distancing is hardly applicable at home, especially in large households but a rapidly respected quarantine at home by the index patient is still useful to prevent COVID-19 transmission within a household [21].

The risk of infection associated with traveling was reduced by travel restrictions and border closures, and that associated with public meetings and events was reduced by a ban on mass gatherings. Nosocomial and workplace infections remained stable over the two periods, other measures such as the use of personal protective equipment having been implemented before lockdown.

This study identifies specific vulnerable populations at high risk of COVID-19 despite lockdown. However, our results should be interpreted with caution given the retrospective design of the study and may not be similar in other settings.

## Conclusion

The lockdown in France effectively contained the COVID-19 outbreak. However, ethnic minorities, vulnerable populations, the elderly, and institutionalized people remained at risk of infection during lockdown. Specific and targeted public health measures have to be implemented to prevent the spread of COVID-19 in these populations. The efficacy of lockdown should also be viewed in light of the collateral effect of prolonged lockdown which may exacerbate socioeconomic inequalities and increase poverty.

## Data Availability

The dataset used and/or analysed during the current study are available from the corresponding author on reasonable request.

## Abbreviations

COVID-19: Coronavirus Disease-19
SARS-CoV-2: Severe Acute Respiratory Syndrome-Coronavirus-2
Vs: versus
